# Investigation of Deformation Pattern and Movement Law of the Huge-Thick Conglomerate Stratum by a Large-Scale 3D Model Test with Distributed Optical Fiber Sensor Monitoring

**DOI:** 10.3390/s21175985

**Published:** 2021-09-06

**Authors:** Qiang Yuan, Jing Chai, Yuzhu Zhang, Yongliang Liu, Yiwei Ren

**Affiliations:** 1 State Key Laboratory of Coal Mine Disaster Dynamics and Control, Chongqing University, Chongqing 400044, China; renyiwei@cqu.edu.cn; 2 College of Energy Engineering, Xi’an University of Science and Technology, Xi’an 710054, China; Chaijing_xust@163.com (J.C.); ylliu.xust@gmail.com (Y.L.); 3 China Coal Technology and Engineering Group, Chongqing Research Institute, Chongqing 400037, China; yzzhang0315@163.com

**Keywords:** HTC, stratum deformation, broken rules, DOFS monitoring, model test, numerical simulation

## Abstract

Mining activities under the circumstances of huge-thick stratum occurrence commonly result in dynamic response of the working face. It is crucial to understand the rock failure and movement of the huge-thick stratum in order to prevent dynamic hazards. This paper introduces distributed optical fiber sensor (DOFS) monitoring into a large-scale model test to investigate the deformation pattern and movement law of the huge-thick conglomerate (HTC); the monitoring results are verified by numerical simulation. The results indicate that DOFS monitoring captures the spatiotemporal evolution of zoning development in the overburden deformation. The deformation field of HTC is illustrated, and there exists a strain basin that can be used to estimate the movement law of HTC. The average strain variability *Ex*, a new homogenization index for characterizing the overburden deformation, is proposed to describe the broken rules of the HTC. The numerical simulation proves the feasibility of the DOFS monitoring method and the correctness of the deformation pattern and movement law. This study provides efficient methods for DOFS monitoring utilization to investigate mining engineering problems and could be beneficial for unearthing the mechanisms of deep ground rock deformation.

## 1. Introduction

An extremely thick and hard rock stratum above the minable coal seam is a special structure of the coal measure stratum and is often encountered in coal mining engineering. It is widely distributed in many mining areas in China, such as the huge-thick conglomerate (HTC) occurrence in the Qianqiu, Changcun, Jiaoping, and Huafeng coal mines [1,2,3,4,5]. The HTCs overlying strata usually have the characteristics of extreme thickness (often more than 100 m) and high compressive strength (uniaxial compressive strength of 50–100 MPa). The mining process of the lower coal seam often leads to unstable movement by the overlying hard stratum, resulting in a large area of suspension under the HTC, high concentration of surrounding rock mining stress and energy concentration, and frequent dynamic load in the mining space. It also leads to extreme mine dynamic disasters such as rock bursts, which introduce hidden dangers to coal mine safety production [6]. The problems associated with safe mining and green mining under these geological conditions have attracted widespread attention [7,8,9,10,11]. To reasonably design a coal mining scheme and establish a detection and early warning system for coal mine dynamic disasters, the study of structural movements and fracture law of HTCs is particularly important.

In recent years, many studies have been performed to investigate problems such as strong coal mine ground pressure, uncommon surface subsidence, and frequent rock bursts caused by the breaking of the thick rock stratum [12,13,14,15,16,17,18,19,20,21]. Table 1 summarizes the research focused on the law of fractured movement of overlying rock stratum and the characteristics of mine pressure behavior under the circumstances of HTC occurrence. This summary proves that physical modelling experiments have played an important role in studying the deformation and movement of the HTC. Unfortunately, no advanced measurement technology has been used to verify the characteristics; traditional monitoring cannot obtain the internal deformation and lacks accuracy. Consequently, these studies cannot provide results with regard to deformation pattern and rock failure rules due to a lack of reliable measurement methods.

Owing to its traits of distributed measurement and suitability to extreme environments, DOFS technology has attracted increasing attention from researchers in the field of mining engineering. Currently, DOFS for mining rock mechanics has been increasingly used in laboratory studies and field applications [32]. For instance, a DOFS network was implemented through borehole drilling to monitor overburden structure evolution [33], internal deformation of the surrounding rock mass [34], and the stability of the coal pillar [35], which significantly enriched the application practice of DOFS in mining engineering. Because the overburden stratum has the characteristics of large area deformation, for which the distributed monitoring of DOFS technology is applicable, this DOFS monitoring research method has become a key approach in field studies measuring, among others, the height of the three zones of the overlying stratum under different mining conditions [36,37], deformation characteristics of the stope roof and roadway floor in coal mines [38,39], the stability of the coal mine surrounding rock mass [40], abutment pressure distribution beyond the working face [41], and roadway supporting safety [42,43]. Moreover, the field installation method, the designation and selection of the optical fiber sensor, and the strain transfer law between the sensor and rock mass are have also been discussed [44] based on field measurement results, and valuable engineering experience has been obtained for further application of DOFS monitoring.

Considering the in-depth application of DOFS technology in mining rock mechanics, the DOFS monitoring method was introduced to study the deformation of HTCs, and some useful results were obtained in a series of studies by the authors’ research team. For example, a displacement measurement device was designed to monitor the internal deformation of the HTC [27,28]. The strain distribution of the whole overburden stratum was revealed [30,45,46,47]. Then, the HTC was considered as the key stratum to investigate its influence on the overburden rock mechanics and the ground surface subsidence characteristics [48,49].

Previous studies have made important progress with respect to the application of DOFS monitoring to geotechnical or geologic engineering, and they have provided an understanding of the ground movement and dynamic hazard prevention under the occurrence of the HTC. Nevertheless, there remain two key scientific issues: (1) engineering limitations determine that conventional methods cannot deeply capture the internal deformation pattern of the HTC, which cannot fully reflect the influence of the HTC deformation that acts on the whole overburden; (2) the DOFS monitoring results provide a better understanding of the broken rules under the impact of HTC deformation; however, owing to the lack of further reprocessing of the data distribution characteristics of the DOFS monitoring, the movement law of the HTC has not been well demonstrated.

Hence, based on previous laboratory experiments and theoretical modeling using Brillouin optical time-domain analysis (BOTDA) technology, and in order to obtain an effective solution for the abovementioned problems, this study carried out a large-scale 3D model test experiment to thoroughly analyze the deformation pattern and movement law of the HTC using the DOFS monitoring method, which has great significance for the popularization and application of DOFS technology for the traditional engineering discipline of mining engineering, and more efficient methods of DOFS system utilization were derived.

## 2. Setup of Large-Scale 3D Model Test and Its DOFS Monitoring System

### 2.1. Basic Principle of BOTDA Technology

**Figure 1:**
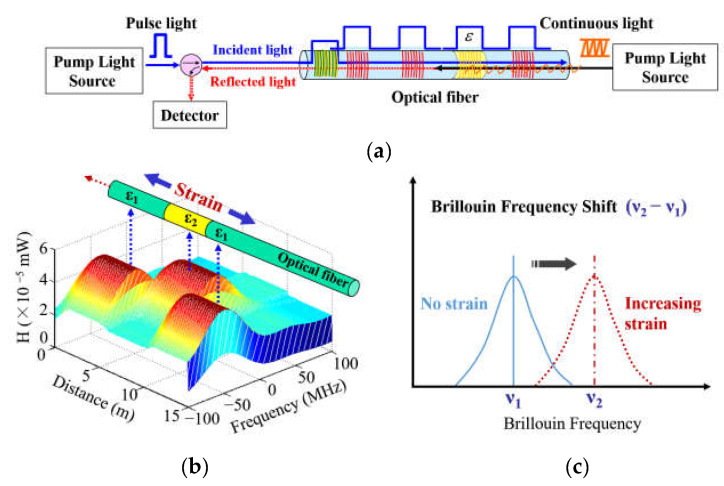
(**a**) Monitoring principle of the both-ends detection of BOTDA; (**b**) the Brillouin frequency shift distribution stimulated by strain change. (**c**) the relationship between frequency and strain.

To study the movement of HTC in physical modeling, BOTDA technology was chosen owing to its high precision and high spatial resolution in indoor experiment measurements. The monitoring principle of the BOTDA-based DOFS system is summarized in Figure 1, which shows that by emitting continuous pulsed light and probe light at both ends of the optical fiber, the Brillouin effect and energy transfer will occur when the wavelengths of the two beams are within a certain range. When the temperature or strain of a certain part of the optical fiber changes, the corresponding Brillouin frequency shift changes accordingly, and the tuning makes the frequency difference between the two beams equal to the new Brillouin frequency shift; then, the Brillouin scattering signal can be received. By detecting the power of continuous light coupled from one end of the optical fiber, it is possible to determine the corresponding frequency difference when the energy transfer reaches the maximum in each small segment of the optical fiber, and the temperature and strain information can be obtained to realize distributed sensing.

### 2.2. Large-Scale 3D Model Test Experiment

The research object of this study was the Qianqiu coal mine in Henan Province, China, where a HTC is located. The engineering geological analysis indicates that the average thickness of the coal seam and the overlying stratum in the Qianqiu coal mine are approximately 15 m and 806 m, respectively. The conglomerate appears from 210 m to 620 m above the coal seam, which is approximately 410 m thick, and was classified into two groups according to the engineering borehole investigation: an upper group and a lower group with thicknesses of 250 m and 160 m, respectively, separated by a weak intercalated layer with a thickness of 1 m. Preliminary field tests show that the conglomerate rock is a typical hard rock, with a maximum elasticity modulus of 32 × 103 MPa and a maximum compressive strength of 75 MPa.

**Figure 2:**
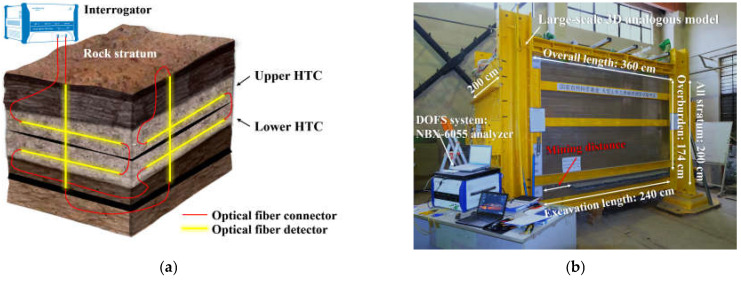
Setup of model test experiment with DOFS monitoring: (**a**) the designate of the large-scale 3D model that demonstrates the optical fiber sensor network and the HTC; (**b**) the physical model and the monitoring system arrangement.

An analogue model test was carried out according to the physical and mechanical properties of the overburden structure in the Qianqiu coal mine. Table 2 shows the overburden structure. A large-scale physical model with a length of 360 cm, width of 200 cm, and height of 200 cm was constructed in the laboratory. The geometric ratio of the physical model and the research object was 1:400, so the analogous model would include a 6 cm high simulated coal seam, a 20 cm high floor stratum, and a 174 cm high overburden stratum, which contains a 100 cm high conglomerate that has a 60 cm thick upper group and a 40 cm thick lower group, as shown in Figure 2a.

Furthermore, direct observation of the overburden deformation was realized by setting a high-strength transparent acrylic board on the front side of the model, which greatly improved the recording of the experimental phenomena. The excavation range of the coal seam was 240 cm in the strike direction and 160 cm in the dip direction. In addition, two working faces were set along the dip direction, resulting in each working face having a width of 80 cm. Given that the experimental physical model was sealed on all sides, there was no entry to the inside of the model for excavation actions; thus, the pull-out simulation method was applied to simulate the coal seam excavation. That is, 60 metal tubes with rectangular cross sections were deployed in the model during the model construction process, each of which had a cross-sectional length of 6 cm and width of 4 cm. This meant that the coal seam had a height of 6 cm and an excavation pace of 4 cm. One metal tube was pulled out from the model when there was one excavation, and 60 excavations occurred in one working face.

Figure 2b also presents the workbench of the DOFS monitoring system, which includes the NBX-6055 optical stress analyzer and 2 mm diameter normal single-mode optical fiber sensors encapsulated in the polyurethane cable of tight buffer fibers wrapped within an elastic protection jacket. Several sets of internal multi-point displacement meters were implanted into the physical model in addition to the optical fiber sensor. To precisely capture the strain distribution as much as possible, the spatial resolution and sampling interval of the optical stress analyzer were set to 5 cm and 1 cm, respectively, which also provided more strain data for a thorough analysis.

### 2.3. Layout of DOFS Monitoring System

**Figure 3:**
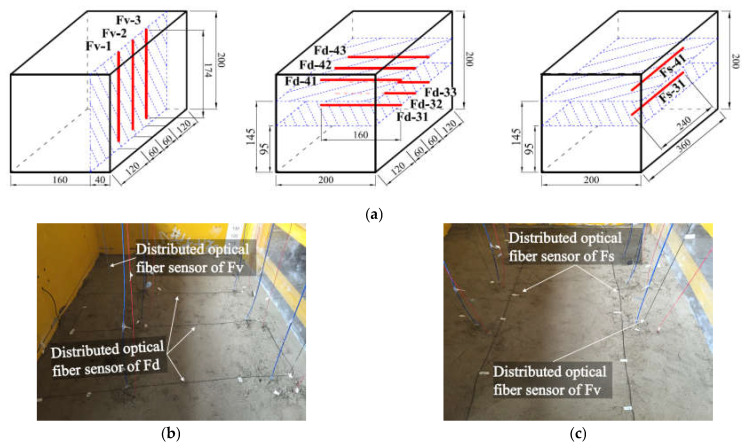
Layout of optical fiber sensors embedded in the conglomerate: (**a**) the spatial location of all the optical fiber sensors embedded in the physical model. Three types of embedding are proposed, which can be addressed as the vertically installed optical fiber sensors Fv1, Fv2, and Fv3; the horizontally installed optical fiber sensor Fd, with the embedding direction of dip (contains six measurement sections); and the horizontally installed optical fiber sensor Fs, with the embedding direction of strike (contains two measurement sections). (**b**,**c**) the installation process of the optical fiber sensor network.

To acquire the omnidirectional monitoring of the HTC rock stratum deformation characteristics, a DOFS network was set up by arranging three groups of optical fibers in three directions of the physical model. One group of optical fibers was installed vertically, namely the optical fiber of group Fv, and the other two groups were installed horizontally. One of the horizontally installed groups was arranged in the strike direction of the model coal seam, and the other was arranged in the dip direction, that is, the optical fiber of groups Fs and Fd. The layout of the optical fiber sensors is shown in Figure 3a.

As shown in Figure 3, there were three vertically embedded optical fiber sensors: Fv1, Fv2, and Fv3. The total length of each Fv sensor was 174 cm, which was equal to the overburden height. The optical fiber sensor Fd contained a total of six sections, deployed in two layers, which referred to the two groups of HTC; Fd31, Fd32, and Fd33 were responsible for monitoring the lower group of HTC, and Fd41, Fd42, and Fd43 were applied for the upper group. Furthermore, the layout of the optical fiber sensor Fs was the same as Fd, with two sensors installed to monitor the two groups of conglomerates. The detailed specifications of the optical fiber sensor installation are shown in Figure 3b,c.

The installation method of the optical fiber sensor can be summarized as follows: the vertically installed optical fibers were fixed on the custom-made device before building the physical model, and they were surrounded by anomalous materials during the construction of the model building. Moreover, each vertical optical fiber was stretched in a straight direction and pre-stressed using a force of 25 N; the horizontally installed optical fibers were embedded while the model was constructed, which means that when the model was built at the specifically designed height, the optical fibers were first inserted into the model, and then the model was built to the next height, until the installation of the five layers of optical fibers was completed.

Finally, it can be concluded that the optical fiber sensors of groups Fv, Fs3, and Fs4, and Fd3 and Fd4 were combined into a network for HTC deformation monitoring. In particular, Fv detected the entire overburden structure, including the HTC, Fs3 and Fd3 were responsible for monitoring the lower group, and Fs4 and Fd4 were responsible for monitoring the upper group.

## 3. Analysis of DOFS Monitoring Results

The simulation excavation was performed for more than a month, with the aim of achieving full monitoring of overburden deformation by the DOFS system. Massive monitoring data in the model test were obtained and provided a thorough explanation of the evolutionary rock failure in the HTC. The DOFS monitoring results analysis of HTC deformation was discussed. More importantly, all the strain data had been already treated with a temperature compensation.

### 3.1. Spatiotemporal Evolution of Overall Overburden Deformation

**Figure 4:**
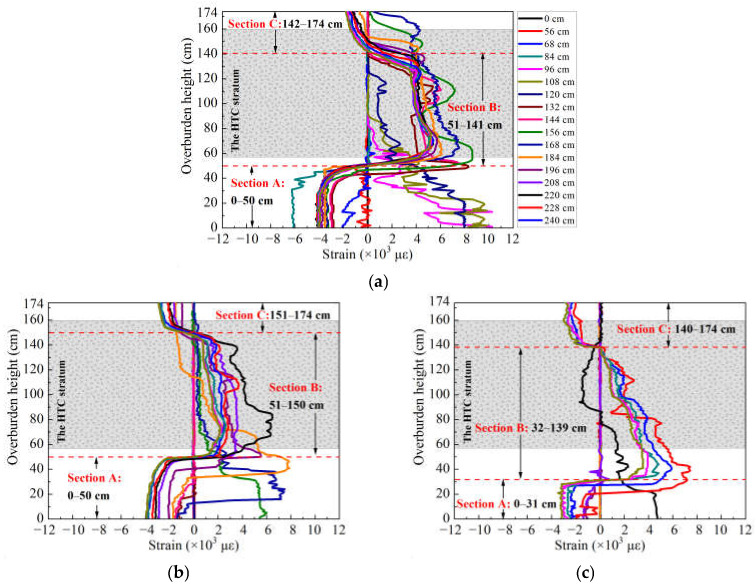
Monitoring results of the optical fiber sensor Fv, which was vertically embedded throughout the whole overburden stratum: (**a**) strain distribution of the optical fiber Fv1; (**b**,**c**) strain distribution of Fv2 and Fv3, respectively. Labels indicate the mining distances.

According to a previous study [48] of overburden deformation based on DOFS monitoring, the strain distribution of vertically installed optical fiber indicates the characterization of overburden deformation in three horizontal areas from the perspective of time and three vertical zones from the perspective of space. Therefore, in accordance with the description of the spatiotemporal evolution law by DOFS monitoring, the measurement results of the optical fiber sensor group Fv are presented to analyze the overall overburden deformation characteristics and distinctly show the shifting of compressive and tensile strain and the stage-form development (see Figure 4). In addition, it should be noted that the monitoring results from only the first working face excavation could be used for the analysis because the overburden stratum that was overlaid on the second working face was significantly affected after the excavation. It was not suitable for analyzing the deformation rules because it was broken before the excavation; therefore, all data presented in this paper were obtained from the monitoring results of the first working face excavation.

Considering the boundary effect and mining influence, the position in which optical fiber Fv1 was embedded should be under full mining effect; hence, the strain distribution of that part of the rock mass would be a better representation of the overburden deformation. As a result, the strain distribution of Fv1 in Figure 4a can be explained from the perspective of time (i.e., increasing the mining distance) as follows.

First, the strain curve changed from zero to a negative distribution, as the working face distance increased from 0–56 cm, which indicates that the measured rock mass is situated in the leading abutment pressure influence area where the increasing compressive stress is concentrated.

Second, the strain curve emerged as a positive distribution that contains multiple stages of development, for a mining distance of 56–84 cm, which indicates that the overburden rock mass has undergone significant deformation and presents varying degrees of movement, resulting in different changes in the distribution at different overburden heights, i.e., the measured rock mass is located in the fractured rock and separation area. However, the strain value is reduced with increasing height, so in the lower stage it is larger than in the upper stage and at least three stages in the strain distribution curve. For instance, taking as an example the strain distribution at a mining distance of 72 cm, the first stage has a height of 25 cm and the second and third stages have heights of 64 cm and 85 cm, respectively, which demonstrates the developed height of the mining-induced overburden vertical three zones based on the characterization pattern mentioned in the previous study; the caving zone increases to 25 cm, and the fractured zone and bending zone are 64 and 85 cm, respectively. Consequently, this illustration laid the groundwork for the regional rock failure analysis and the vertical three-zone identification.

Finally, the measured rock mass will be in the recompacting area when the working face advances over 84 cm, according to the characterization pattern, and the rock mass is subjected to compressive stress again under the influence of overburden deformation so that the strain distribution would restore and maintain a negative value. However, from Figure 4a, it is evident that only part of the overburden rock mass is maintained in a compressive stress state, and most of the overburden stratum is still under tensile stress; specifically, the tensile strain is distributed in and spreads over the HTC.

On the other hand, from the perspective of space development (i.e., increasing the height of overburden deformation), the strain distribution can be presented as follows: for a model height ranging from 0–50 cm (section A in Figure 4a), the strain curve develops as a negative stage form, which indicates that the measuring rock stratum is in a state of compressive stress, and it is the characterization of recompacting of rock mass in the caving zone. For a height ranging from 51–141 cm (section B in Figure 4a), the strain distribution presents as a wave-like corrugation, which can be divided into two wave peaks. The first wave peak has a strain of 7000 με and is located at a model height of 60 cm, while the second wave has a smaller strain of 5000 με. The corrugation shape of the strain distribution means that for a certain range of overburden stratum that is still situated in tensile stress, the rock mass could be moving downwards or overhanging above the mining gobs. When the height is over 141 cm (section C in Figure 4a), the strain distribution returned to negative and remained stable.

### 3.2. Strain Distribution of the Lower Group of HTC

**Figure 5:**
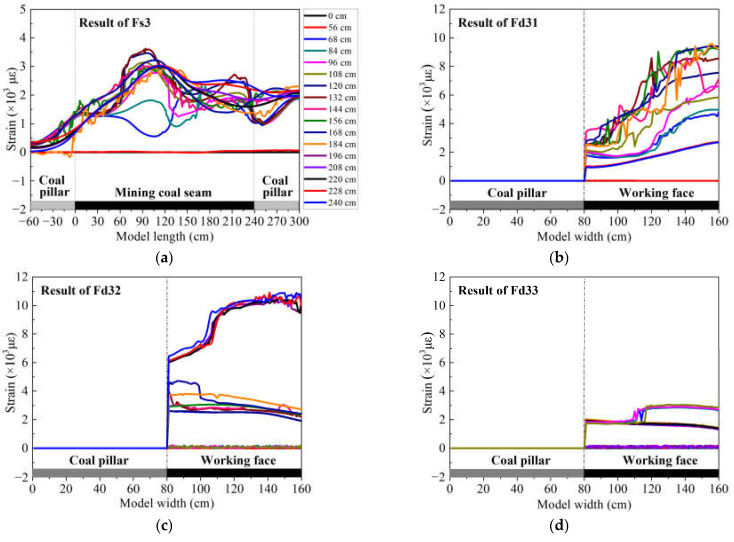
DOFS monitoring results of the lower group of HTC: (**a**) measurement result of optical fiber sensor Fs3, which indicates the strain distribution of the lower group of HTC at the direction of strike; (**b**–**d**) measurement results of optical fiber sensors Fd31, Fd32 and Fd33, respectively, which indicate the strain distribution of the lower group of HTC at the direction of dip.

The optical fiber sensors Fs3 and Fd3 are embedded in the lower group of the HTC, and the monitoring results of those sensors represent the deformation of the lower group conglomerate. Fd3 contains three measurement sections: Fd31, Fd32, and Fd33. Figure 5 shows the strain distribution of each optical fiber sensor.

Figure 5a presents the strain distribution of optical fiber sensor Fs3, and it shows that the strain distribution of optical fiber Fs3 presents a “humping shape”, which has a maximum strain of 3586.65 με and is located at a mining distance of 100 cm. This implies that the lower group of HTC is bending downward on the whole and has a rotary motion, and the lower group stratum has not been broken. In addition, the strain curve swells up at a mining distance of 220 cm, which means that the rock mass has been subjected to tensile stress in that area. There was a sawtooth shape on the strain curve at mining distances ranging from 108–144 cm near the humping shape peak, which indicates that there is a microfracture in the stratum in that area.

Figure 5b–d are the strain distribution of optical fiber sensors Fd31, Fd32, and Fd33, respectively. From these results, it can be seen that the variation in the strain is not quite the same as that of Fs3, forming an arched curve that appears like a non-fully developed humping shape; the strain starts to increase at the model width of 80 cm and does not decrease at a model width of 160 cm. Considering that the front side of the physical model did not design the coal pillar, the stratum material touched the model frame (i.e., the acrylic plate) smoothly. Therefore, the deformation could be enlarged at a model width of 160 cm. Nonetheless, this strain curve, which has an inconspicuous humping shape, shows that the lower group of HTC has not immediately broken in the direction of the dip, and the rock stratum could have a rotary motion that starts at a model width of 80 cm. From the embedded position of the three optical fiber sensors of group Fd, Fd31 is located 60 cm before the starting working face, so its strain distribution is basically unchanged at a mining distance of 0–48 cm, and the rock mass adjacent to Fd31 will impose a mining influence until the mining distance is 56 cm, which results in a strain variation of Fd31. The strain distributions of the optical fiber sensors Fd32 and Fd33 changed after mining distances of 108 cm and 168 cm, respectively. In addition, the maximum strain of Fd31 and Fd32 is approximately 20,000 με, which signifies that the deformation status is consistent in the same stratum, and Fd33 may be undergoing a boundary effect so that the strain value does not reach the value that should be reached.

### 3.3. Strain Distribution of the Upper Group of HTC

**Figure 6:**
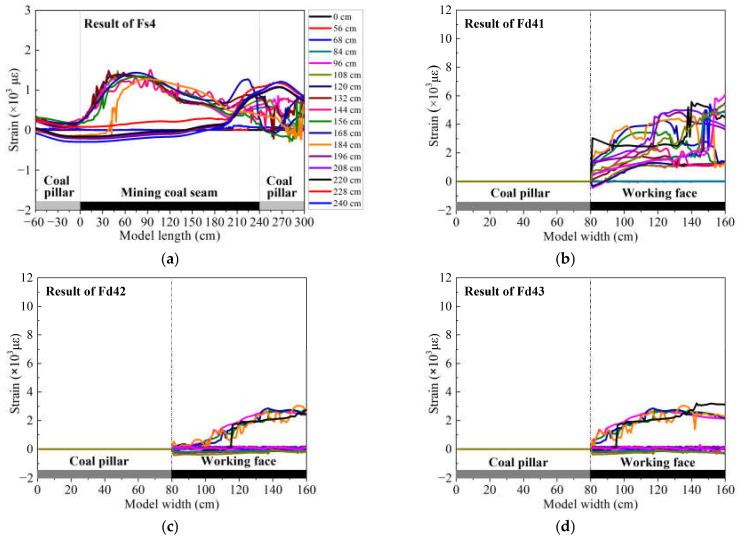
DOFS monitoring results of the upper group of HTC: (**a**) measurement result of optical fiber sensor Fs4, which indicates the strain distribution of the upper group of HTC at the direction of strike; (**b**–**d**) measurement results of optical fiber sensors Fd41, Fd42 and Fd43, respectively, which indicate the strain distribution of the upper group of HTC at the direction of dip.

The optical fiber sensors Fs4 and Fd4 are embedded in the upper group of the HTC, and the monitoring results of those sensors are the expression of deformation and movement of the upper group of the HTC, as shown in Figure 6.

Figure 6a shows the strain distribution of optical fiber sensor Fs4; it is evident that the strain curve has the same characteristics as Fs3. The humping shape change and sawtooth form were still observed, but only the location of the hallmark of those strain distributions differed in the monitoring results of both Fs3 and Fs4. The characteristics of strain distribution of Fs4 appear later than that of Fs3, which means that the higher upper group of HTC will break after the lower group of HTC. It can also be found that the maximum strain of Fs4 is reduced to 1425.15 με, which is half that of Fs3 and is evidence that the degree of deformation of Fs4 is smaller than that of Fs3.

From Figure 5a, it should also be noted that the strain distribution of Fs4 presents two changes. First, there is a humping shape when the mining distance is 0–184 cm, which is consistent with Fs3. However, the second change is that the strain value becomes negative after a mining distance of 184 cm, which means that the stratum has been subjected to a compressive stress in the area with a model length of 0–180 cm, and it then recovered to the tensile stress state in the area of 180–240 cm.

Figure 6b–d show the strain distributions of the optical fiber sensors Fd41, Fd42, and Fd43, respectively. The strain variation changes as the mining distance increases. The strain curve of Fd4 has a consistent increasing tendency with Fd3; the arched curve, without an obvious peak in the strain value and the sawtooth, is located at the model width of 90–110 cm. However, the maximum strain of the three measurement sections of the optical fiber sensor Fd4 is approximately 40,000 με, which is less than that of Fd3, indicating that the deformation and movement of the upper group of the HTC is much smaller than that of the lower group. This strain distribution comparison of Fd3 and Fd4 follows the same rule as that of Fs3 and Fs4, as mentioned above.

From the perspective of mining overburden broken mechanisms and morphological characteristics, the rock stratum will usually bend downward with a rotary motion under the influence of mining. This phenomenon has been proven by the strain distribution of DOFS monitoring results, such as the monitoring results analysis of optical fiber sensor Fs, in this study and other previous studies. However, the rock stratum in the dip direction of the working face usually does not form obvious characteristics of broken line development in the strike direction because of the small suspension length, and it often bends and sinks by multi-fracture segmentation or rotary movement based on the boundary coal pillar. In this study, these characteristics have been verified by the monitoring results of the optical fiber sensors Fd3 and Fd4, which indicates that there will be multiple microfractures generated in the HTC on the side of the working face. The cracks will not intersect through the rock mass because of the limited stratum thickness, and the HTC will rotate and move to the gob in the form of a multilayer or whole layer in the position where the crack is opened. Moreover, the deformation and movement differences of the upper and lower groups of the HTC, namely the lower group of strata, have a relatively higher response to mining activities, and the deformation of the upper group usually lags behind that of the lower group.

## 4. Characterization of the HTC Deformation

### 4.1. Zoning Development of Overburden Stratum

**Figure 7:**
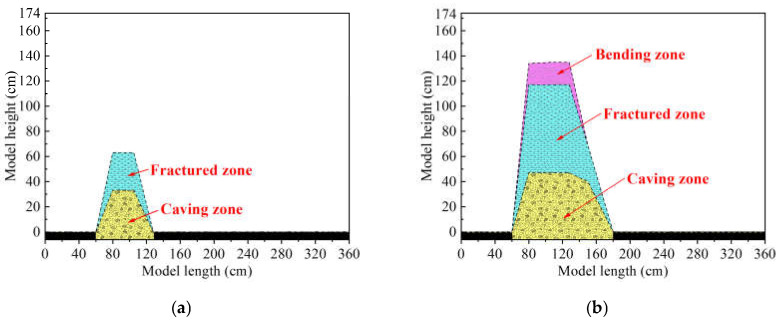

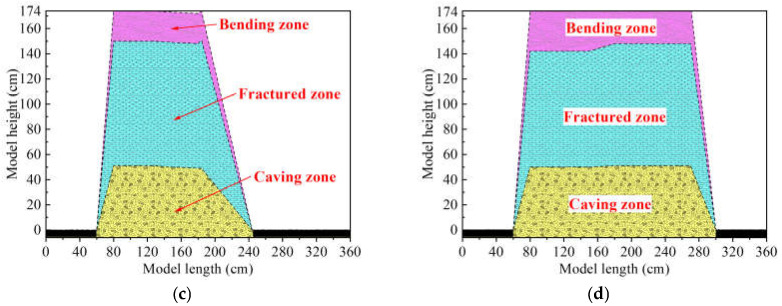
Development of the vertical three zones of overburden deformation: (**a**) shows that only two zones have been formed when the mining distance is 68 cm; the mining influence does not have enough impact on the higher rock stratum so that the bending zone cannot be created; (**b**) shows that the bending zone has emerged when the mining distance is 120 cm; (**c**) shows that the broken rock stratum has developed up to the full height of overburden when the mining distance is 184 cm; (**d**) shows the overall development of vertical three zones when the excavation is accomplished.

According to the previous research results of the characterization of overburden deformation with DOFS monitoring [49], the characterization rules of the zoning development of the mining overburden stratum (i.e., the vertical three-zone development) can be summarized as follows: the height of the caving zone, fractured zone, and bending zone are consistent with the height of the first, second, and third stages of the strain distribution, that is, sections A, B, and C in Figure 4. As a result, the vertical three-zone development of the overburden stratum can be extracted based on the monitoring results shown in Figure 4. Combining the different zone heights and the different mining distances, the overburden zoning development is shown in Figure 7; the broken area is also illustrated.

Figure 7a shows the vertical three-zone development of the overburden deformation when the mining distance is 68 cm; the broken area of the mining overburden presents a positive trapezoid shape, which includes a caving zone and fractured zone with an average height of 30 cm and 60 cm, respectively. The bending zone was not created because the failure height of the rock mass was just as high as that of the lower group of HTC, and the overlying hard and thick conglomerate would not easily bend or collapse. In fact, it is difficult to produce a bending zone in such a rock mass with the mechanical property of high rigidity; only a massive collapse or caving of rock mass below the HTC, gravity, and mining-induced stress would be sufficient to bend that stratum.

Figure 7b shows the vertical development of the overburden deformation for a mining distance of 120 cm, where half of the coal seam was excavated. With the increasing area of overburden stratum broken under the mining influence, three integrated vertical zones were produced, and the heights of the zones were 45 cm, 115 cm, and 135 cm. It can be concluded that when the mining influence impacts the rock mass near Fv2, more rock mass starts to participate in overburden deformation and induces massive movement of overburden strata, which initiates the deformation of the HTC. Finally, this series of actions prompted the emergence of a bending zone.

Figure 7c shows the vertical three-zone development of the overburden deformation with a mining distance of 184 cm. It can be observed that the broken area spread to the entire overburden. The maximum height of the caving zone was enlarged to 50 cm; it gradually decreased along the strike direction of the coal seam and ended at a model length of 240 cm. In the same way, the height of the fractured zone and bending zone are also subjected to the mining influence; they will decrease as the working face increases. The overburden was caused by a strong deformation and movement, and the heights of the fractured and bending zones are approximately 145 cm and 174 cm, respectively.

Figure 7d shows the vertical three-zone development of the overburden deformation when the mining distance is 240 cm; with the coal seam excavation accomplished, the final integrated three-zone structure of overburden deformation is formed under full mining activities. The height of the fractured zone was slightly decreased compared with the broken area of the overburden stratum at a mining distance of 184 cm, whereas the height of the bending zone increased slightly, which means that the overburden load was adequately transferred to the gob. Finally, the vertical three-zone development can be addressed as follows: a 50 cm height of the caving zone, a 140 cm height of the fractured zone, and a 174 cm height of the bending zone.

According to the above analysis, it was found that the height of the caving zone first increased and then decreased as the working face advanced in the strike direction of the coal seam. This kind of change indicates that the overlaying load will apply compressive stress on the rock mass within the caving zone, which results in a decrease in the height of the caving zone; the height of the fractured zone increases rapidly during the initial stage of excavation and then decreases after the excavation finishes, which is the response of rock mass compression in the later stage of overburden deformation. The height of the bending zone is less than that of the caving zone, and it will develop to the ground surface after the working face advances a certain distance. This rule of zoning development based on DOFS monitoring results is consistent with mining rock mechanics and overburden deformation theory in mining engineering.

In conclusion, the zoning characteristics of overburden deformation based on DOFS monitoring results have obtained the dynamic development law of the three vertical zones, and the deformation of the overburden stratum and the broken range of the three vertical zones will be through a dynamic process under the mining influence. This shows that after the coal seam under the HTC is excavated, the height of the caving zone is usually more than eight times that of the mining height of the coal seam, and the hard HTC forms the main key stratum. Therefore, the sub-key stratum, which is located at a overburden height of 30 cm, plays a negligible role of the key stratum, and it will fracture and collapse immediately with the breaking of the immediate roof stratum; in other words, the whole rock stratum below the HTC will constitute the caving zone, which has a height of 50 cm. In the meantime, the entire HTC would form the fractured zone, and the rock stratum above the conglomerate forms the bending zone.

### 4.2. Deformation Field of the HTC

**Figure 8:**
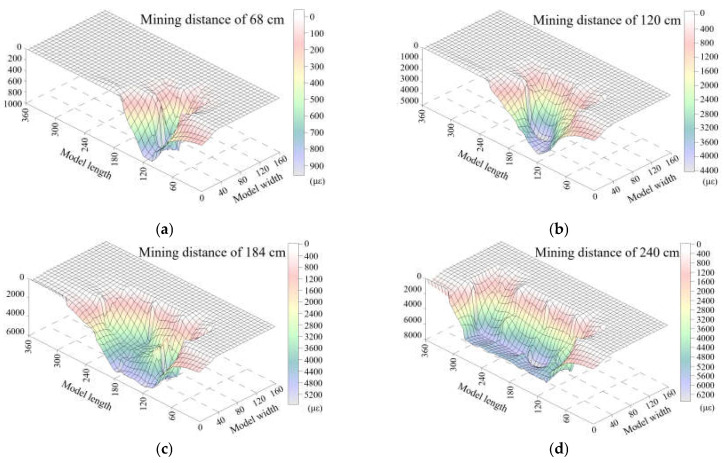
Deformation field of the lower group of HTC at the different mining distances in the model test based on DOFS monitoring characterization.

**Figure 9:**
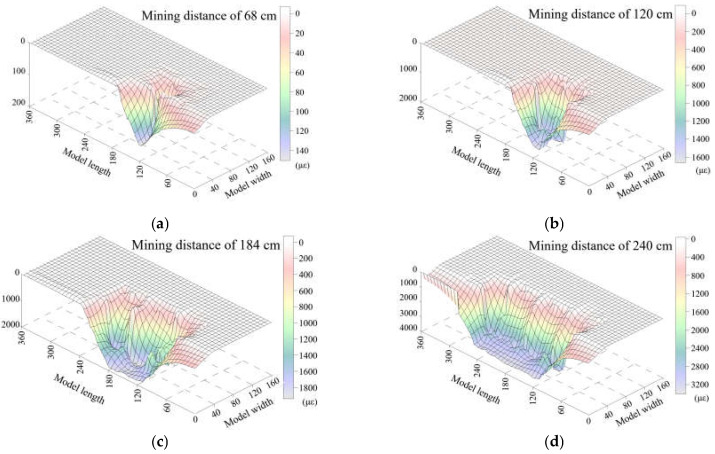
Deformation field of the upper group of HTC at the different mining distances in the model test based on DOFS monitoring characterization.

A total of ten measurement sensors were horizontally embedded in the HTC according to the embedding layout of the optical fiber sensors, five of which were in the lower group, and the others in the upper group. Therefore, a deformation field was calculated based on these five sensors by the surface fitting of five strain distribution results, which are shown in Figure 8 and Figure 9 for the deformation field of the lower and upper groups, respectively. This approach uses the strain distribution (here, the positive value of strain means that the rock mass is in a tensile state and breaks downward) to reveal the deformation status of the stratum and attempts to characterize the HTC deformation based on DOFS monitoring, which offers more suitable methods for describing the structural characteristics.

Figure 8 shows the deformation field of the lower group of HTC, which shows that the degree of deformation is small and only has a maximum strain of 900 με when the mining distance is 68 cm. This means that the lower group of conglomerates is in a state of slight deformation; the deformation will increase to a maximum strain of 40,000 με when the mining distance is 120 cm, which means that the lower group of the conglomerates has been fractured and starts to move downward. The deformation field creates a strain basin when the mining distance is 184 cm, and it is located in the model lengths ranging from 60–120 cm. This indicates that the 60–120 cm range of the lower conglomerate group has a large deformation and movement, and the basin will be completed after mining to 240 cm. This basin shape feature of the deformation field means that the rock stratum has been broken and collapses downward to the gob. If there is no deformation basin, it should only exhibit a slight movement (or micro fracture) and has no large deformation. Therefore, it can be concluded that the lower group of HTC will be broken in the whole stratum after the excavation of 184 cm.

Figure 9 shows the deformation field of the upper group of the HTC, which clearly indicates that the deformation and movement of the upper group is smaller than that of the lower group. There is almost no change during the excavation of 68 cm. Then, the rock stratum undergoes a slight deformation until the mining distance is 120 cm. Up to 184 cm, the rock stratum will fracture and still maintain a small deformation; that is, the upper group of the HTC will stay in the original horizon without a large fracture or movement, and finally, the upper group of the conglomerate will break and collapse when the mining distance is 240 cm.

From the scope of the strain basin of the deformation field, it can be found that the deformation of the upper group of the HTC is based on the movement of the lower group of the HTC, and the states of the two moving subsidence basins are the same. The strain basin of the upper group is slightly smaller and also shows that both groups of conglomerates have the characteristics of synchronous deformation.

As a result, the surface fitting of the strain distribution of DOFS monitoring was obtained and demonstrated the deformation field variation of the two groups of HTC; more importantly, the distribution law of the internal deformation and movement of the rock stratum was revealed in an intuitive way. Thus, the deformation of the rock stratum can be distinguished by analyzing the strain basin characteristics, which provides a basis for the further analysis of the broken rules of the HTC.

### 4.3. Broken Rules of the HTC

Given that the strain distributions of the optical fiber sensors Fs and Fd represent both groups of HTC, and according to the average frequency shift theory proposed in reference [50], this study creates a homogenized treatment to use the strain distribution to characterize the overburden deformation. Therefore, the average strain variability is proposed, and it can be calculated using the following equation:

(1) $$E_{x}={\left(\frac{1}{n_{1}},\sum_{i=1}^{n_{1}}\varepsilon_{i}\right)}_{1}+{\left(\frac{1}{n_{2}},\sum_{i=1}^{n_{2}}\varepsilon_{i}\right)}_{2}+\cdots +{\left(\frac{1}{n_{f}},\sum_{i=1}^{n_{f}}\varepsilon_{i}\right)}_{m}$$

where *Ex* is the averaged strain variability, *n_f_* is the sampling number of one measurement of the optical fiber sensor, which is determined by the sampling interval, *ε_i_* is the strain value of each sampling point, and *m* is the number of optical fiber sensors that are embedded in the targeted monitoring object.

**Figure 10:**
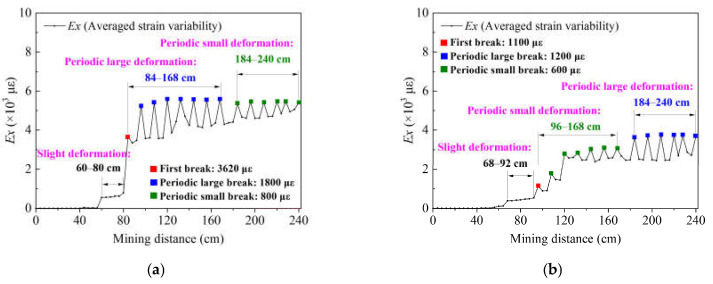
Broken rules of the HTC characterized by DOFS monitoring results: (**a**) averaged strain variability of the lower group conglomerate; (**b**) averaged strain variability of the upper group conglomerate.

The physical significance of *Ex* is that it represents the degree of deformation of the overburden rock stratum; a larger *Ex* indicates a higher degree of deformation, i.e., the stratum will be broken more violently with a larger *Ex*. Therefore, the stratum will have a larger deformation when *Ex* presents a larger change as the mining distance increases. In other words, the roof weighting (the first weighting or periodic weighting) must induce a large *Ex* variation based on the mine ground pressure control theory. Consequently, *Ex* can be used to determine the broken rule of the HTC, which can be summarized as follows: a step offset of *Ex* represents a larger deformation or large breakage of the HTC, and the *Ex* value indicates the degree of that deformation. Figure 10 shows the *Ex* variation calculated by the monitoring results of the optical fiber sensors embedded in both groups of HTC. Figure 10a shows the *Ex* results calculated by the strain distribution of Fd3 and Fs3, which also represents the broken rules of the lower group of HTC, and Figure 10b shows the *Ex* results calculated by the strain distribution of Fd4 and Fs4, which also represents the broken rules of the upper group of HTC.

As shown in Figure 10a, the broken rule can be summarized into three stages: the first stage is when the mining distance is between 60 cm and 80 cm. In this stage, the lower group of HTC has begun to undergo a weak deformation; the deformation is relatively small and is maintained at a certain level with the excavation. The second stage is when the mining distance is between 84 and 168 cm; the *Ex* curve appears to have a sawtooth multi-peak shape, and the average sawtooth peak value is about 1800 με, which indicates that the lower group of HTC is in the stage of periodic large deformation. That is, the rock strata break periodically with the advance of the working face, resulting in the periodic weighting of the working face. The third stage is when the mining distance is between 184 and 240 cm; the *Ex* curve still shows the sawtooth shape, but the value of the average sawtooth peak decreases to 800 με, which indicates that the deformation state of the lower group of HTC tends to be eased. However, it still maintains the motion state of periodic small deformation.

The broken rules of the upper group of the HTC also have three stages, as shown in Figure 10b. The first stage is the mining distance of 68–92 cm, where there is only slight deformation; the second stage is the mining distance of 96–168 cm, and the *Ex* curve also appears to have a sawtooth shape with a tooth peak of 600 με, which indicates that the upper group of HTC is in a periodic small deformation; the third stage is the mining distance of 184–240 cm, where the sawtooth shape remains and there is a tooth peak of 1200 με, which indicates that the stratum has a large periodic deformation.

Based on the ground pressure control theory, the first break of the lower group of HTC is located at a mining distance of 84 cm, after which it will experience 13 periodic breaks; the upper group of HTC has the first break at the mining distance of 96 cm, and has 12 periodic breaks. Furthermore, from the change tendency of the *Ex* curve, it can be found that the *Ex* curve rises as the mining distance increases, and it remains stable at a mining distance of 184 cm, which means that the lower group of HTC still exhibits a trend of continuous deformation when the excavation is completed. However, the upper group of HTC maintains a small deformation until the excavation of 184 cm, and it then undergoes a large deformation until the end.

Both analyses of broken rules of the two groups of HTC show that the *Ex* variation of the lower group is larger than that of the upper group, which proves that the overburden deformation will decrease with increasing stratum height. Meanwhile, there is a transition of the deformation status in the *Ex* variation of both groups of strata, such as the lower group first presenting periodic large deformation, after which it changes to a periodic small deformation, and the upper group presents the inverse situation. This transition means that the deformation of the lower group is relatively severe in the early stage (84–168 cm) of excavation, and the deformation has been gradually limited with the continuous accumulation of broken rock mass below the lower group of the HTC. This results in the stratum presenting only a periodic small deformation in the later stage of excavation (184–240 cm). However, the upper group of the HTC did not produce a large deformation at the initial stage of the excavation. The upper group began to undergo large deformation only when the lower group was broken on a large scale.

From the results, it can be concluded that the broken distance of the lower group is about 84 cm of excavation, and it is 184 cm for the upper group; in other words, the lower group of HTC will collapse at a mining distance of 84 cm, and the upper group will collapse at a mining distance of 184 cm. More importantly, from the *Ex* variation, the broken rule can be drawn as follows: the lower group of HTC will undergo a slight deformation for a mining distance of 0–84 cm. Then it will be broken to collapse at a mining distance of 84 cm and maintain a large periodic deformation until the mining distance is 184 cm; finally, it will transition into a small periodic deformation because of the mining influence.

However, the deformation of the upper HTC group depends on the deformation law of the lower group. The deformation of the upper group will undergo a slight deformation at a mining distance of 0–68 cm, then the small deformation will accumulate at a mining distance of 68–92 cm, and it will be broken to collapse at a mining distance of 96 cm. After that, there will be a small periodic deformation for a mining distance of 96–184 cm, which changes into a large deformation at a mining distance of 184 cm, until the excavation is accomplished.

## 5. Discussion of Feasibility of DOFS Monitoring Method

Considering that the research object of this paper has the obvious attribute of a “black box” problem, that is, it is usually impossible to explore and examine the results intuitively, the numerical simulation could be another key approach to study this kind of issue. In fact, numerical simulation for underground mining has been widely utilized in the last decade. In the study of rock fracture evolution, three basic experiments involving rock uniaxial compression, uniaxial tension, and shear fracture using numerical simulation [51] were conducted to obtained nonlinear stress–strain curves and three-dimensional images of rock fracture evolution. The finite difference code and distinct element code were coupled to investigate the AE characteristics of the Kannagawa underground powerhouse cavern in Japan [52]. For the numerical simulation methodology, the Fish language of FLAC 3D was utilized to simulate different row spacing optimization schemes [53]. Finite element analysis technology utilizing hydraulic-mechanical-damage coupling and the adaptive finite element–discrete element method was proposed to simulate multistage propagation of 3D multiple hydraulic fractures [54,55]. In coal mining–induced rock mechanics research, the failure and deformation characteristics of roadways at inward locations in the lower coal seam were investigated by FLAC 3D to realize the rational location of roadway arrangements [56]. Simulating the static and dynamic behavior of coal pillars using FLAC 3D was proposed to examine the mechanisms involved in pillar failure as well as the pillar’s dynamic response [57]. The stability of mining roadways with 6.0 m section coal pillars under the influence of repeated mining was analyzed by FLAC 3D modeling [58]. The evolution of mining-induced stress and fracturing during the mining process in multilayered heterogeneous rock strata was studied by a continuum-based discrete element method [59], and the time-dependent strength and deformation as well as the influence of loading ratio and pillar shape on the long-term strength of rock pillars have been studied [60].

Therefore, the deformation of the HTC has been investigated by numerical simulation. The feasibility and accuracy of the DOFS monitoring method is discussed by comparing the vertical displacement field of both groups of HTC acquired by numerical simulation and the strain field, which is obtained by DOFS monitoring.

**Figure 11:**
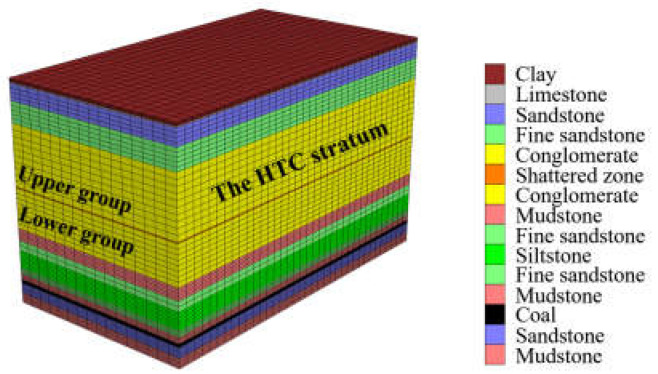
Numerical modeling of the overburden stratum that contains two groups of HTC by FLAC 3D.

The deformation law of the HTC under a mining influence was simulated using FLAC 3D. The geological conditions of the Qianqiu coal mine are shown in Table 3, and the numerical model is shown in Figure 11. The numerical model is consistent with the study procedure of the large-scale 3D model test. The numerical simulation is in agreement with engineering practice, which means that the numerical modeling is derived from field geological conditions.

**Figure 12:**
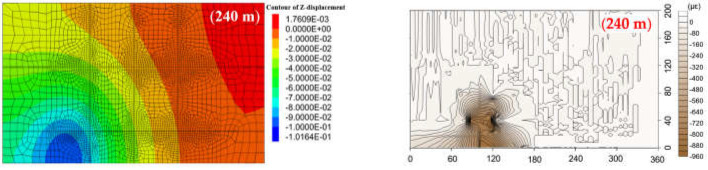

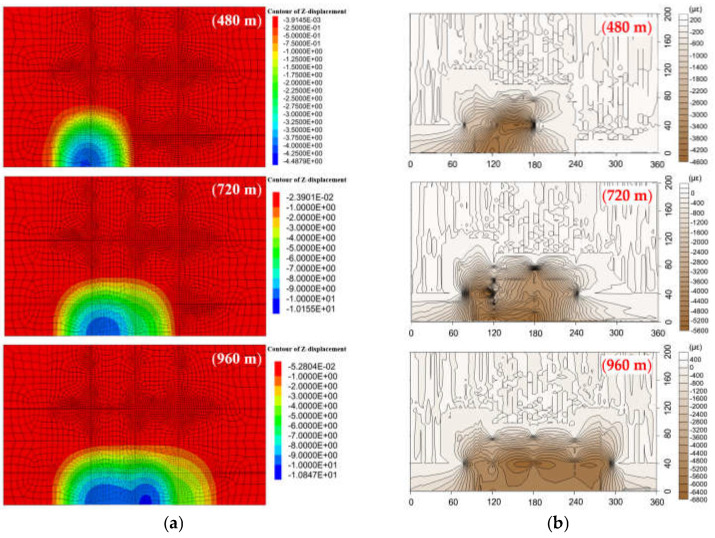
Vertical displacement distribution and comparison of the lower group of HTC strata: (**a**) the displacement field obtained by numerical simulation at the different mining excavation distances; (**b**) the deformation field calculated by DOFS monitoring results at the corresponding excavation distances.

**Figure 13:**
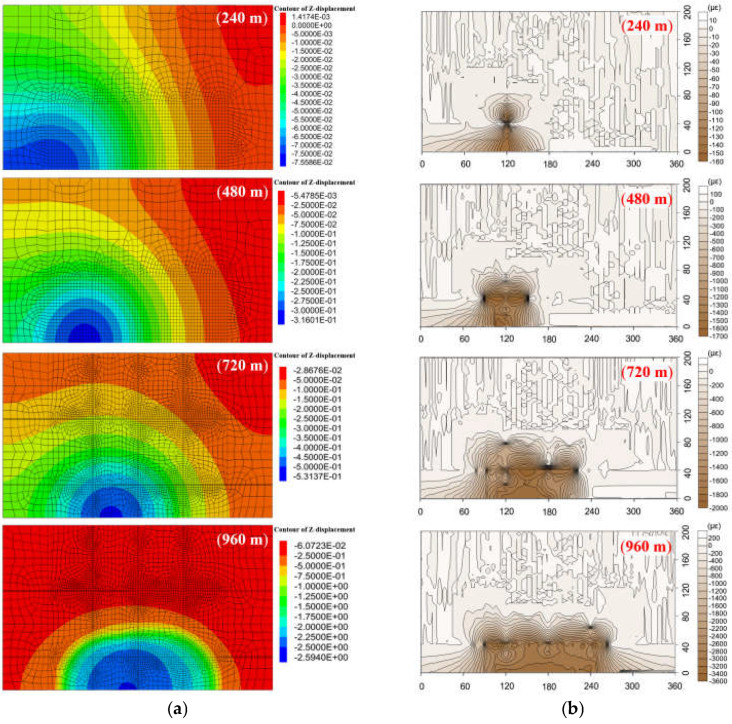
Vertical displacement distribution and comparison of the upper group of HTC strata: (**a**) the displacement field obtained by numerical simulation at the different mining excavation distances; (**b**) the deformation field calculated by DOFS monitoring results at the corresponding excavation distances.

The numerical simulation procedure complied with the standard manual [61,62,63] and can be summarized as follows: a numerical model 1440 m in length, 800 m in width, and 886 m in height was built according to Table 3. The model had 286,498 network units and a total of 351,648 node units. The excavation face was 15 m in height, 400 m in width, and 960 m in advancement length, and 240 m boundary coal pillars were set on both sides of the model. The fifteen simulated rock strata were contacted to each other by attaching mesh, so that fourteen interfaces were set up between each stratum layer. Then, the mechanical parameters of each interface were determined according to the adjacent rock strata clamping the interface; the strength of the interface was usually set as ten times smaller than the adjacent rock strength, based on the manual of FLAC simulation. Elasto-plastic stress analysis was performed to calculate the stress and deformation at each mining distance. The material model was based on the Mohr-Coulomb failure criterion. Supporting clamps were applied to the bottom face and four surrounding faces of the model, meaning that those faces had no speed and displacement. The top face was set free. The large deformation mode was used in the FLAC calculation to match the movement of the rock stratum. The comparison results of the vertical displacement field where the optical fiber sensor was located in the lower group of the HTC and the strain distribution during the four excavation stages are shown in Figure 12 and Figure 13, respectively. The strain distribution indicates the vertical deformation of the rock stratum. Figure 12a shows the vertical displacement field of the rock stratum, and Figure 12b shows the corresponding deformation field obtained by DOFS monitoring.

From Figure 12, the maximum displacement of the lower group of HTC at different mining distances is fitted with the maximum value of the corresponding strain of DOFS monitoring. When the working face was increased to 240 m, the maximum vertical displacement of the HTC in the lower group was 0.10 m. The whole rock stratum was not damaged, the corresponding maximum strain was 960 με, and the range of displacement values tested by the optical fiber and physical model was basically the same. The working face was excavated to 480 m, 720 m, and 960 m, and the maximum vertical displacement of the lower group of the HTC was 4.49 m, 10.15 m, and 10.85 m, respectively; the corresponding maximum strain values were 4600 με, 5600 με, and 6800 με, respectively.

From Figure 13, the maximum vertical displacement of the upper group of the HTC is approximately 0.075 m when the mining distance is 240 m, and the strain variation is 160 με, which indicates that the HTC is basically not deformed at this time. When the working face is excavated to 480 m, 720 m, and 960 m, the maximum vertical displacement of the HTC was 0.309 m, 0.53 m, and 2.59 m, and the corresponding maximum strain variation was 1700 με, 2000 με, and 3600 με, respectively. Thus, this shows that the upper group of HTC will be significantly broken when the mining distance exceeds 720 m.

**Figure 14:**
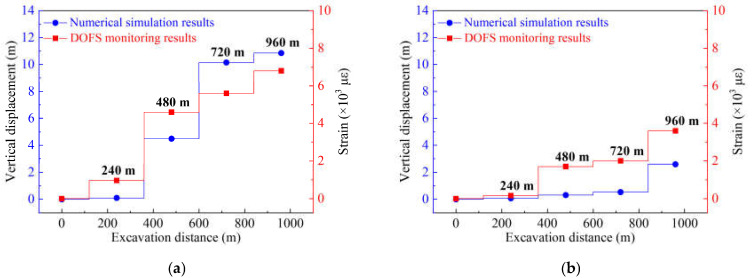
Comparison of the maximum displacement of HTC obtained by numerical simulation and DOFS monitoring: (**a**) the maximum displacement of the lower conglomerate at different mining distances; (**b**) the maximum displacement of the upper conglomerate at different mining distances.

According to the analysis above, the results of the numerical simulation and DOFS monitoring were compared and are shown in Figure 14, where the maximum displacement of the HTC and the maximum strain are illustrated to explain the consistency of the numerical simulation and DOFS monitoring. The variation in the results of both methods is basically the same; there is the same varying trend of the maximum displacement and the strain variation, as well as a good linear relationship between them. In addition, Figure 14 shows that the degree of deformation of the lower group of HTC is larger than that of the upper group, the movement law of the upper group of the HTC is controlled by the lower group, and both groups of strata have a collaborative deformation.

In conclusion, the range of the deformation field and the change in displacement of the HTC in the upper and lower groups show the same change in the numerical simulation and DOFS monitoring results, which proves that the DOFS monitoring results are reliable. Moreover, by comparing the results of both methods, it can be found that the large deformation of the upper group of the HTC occurs after an excavation of 720 m, and that of the lower group occurs after an excavation of 240 m. If the field data of the numerical simulation are converted to model test data using the physical modeling principle (a geometric similarity constant of 1:400), the mining distance to the collapse of the lower and upper groups of the HTC is after 60 cm and 180 cm, and the same determination distance calculated by *Ex* is 84 cm and 184 cm, respectively, which means that the broken distance is nearly the same. Consequently, the broken rule obtained by numerical simulation is consistent with the calculation results of the averaged strain variability *Ex*, which further proves the correctness of the DOFS monitoring results.

## 6. Conclusions

This paper introduced the DOFS monitoring method in mining engineering to reveal the deformation pattern and movement law of the HTC by implementing a large-scale 3D model test, and the feasibility of the DOFS monitoring method was verified by numerical simulations. The results not only demonstrate the comprehensive spatiotemporal evolution process of the fracture and breakage of the HTC, but they also provide an effective solution for probing the inner mechanism of rock mass failure, which cannot be obtained specifically by the conventional detection method. It would be beneficial to study the field engineering roadblocks and basic scientific issues related to deep ground engineering. The detailed conclusions are as follows.

(1) The strain distribution acquired by the vertical optical fiber sensors shows that the tensile stress will remain in the HTC after the excavation is complete, which is unlike the common soft sedimentary rock, where the compressive stress occurs when the mining is over. The vertically embedded sensors proved that microcracks were generated in the HTC, and the stratum moved downward by the fractures in a rotary motion into the mining gob.
(2) The dynamic development of the three vertical zones demonstrates the broken area of the HTC; the caving zone is much larger than the usual zone. The hard HTC forms the main key stratum, which causes the fracture zone to spread over the conglomerate area, and the stratum above that will bend over.
(3) The deformation field of the HTC was deduced by the surface fitting of the strain distribution of the DOFS monitoring, which provided an intuitive way to detect the internal deformation and movement law of the rock stratum. Specifically, the strain basin shape of the deformation field was identified to describe the broken status so that the broken law can be distinguished through the analysis of the strain basin characteristics.
(4) The concept of average strain variability was proposed to determine the broken rules of the HTC. The calculation of the *Ex* variation indicates that the broken distance of the lower and upper groups is 84 and 184 cm of the excavation, respectively, and the deformation of the upper group depends on that of the lower group. The broken rules based on the *Ex* variation can be summarized as follows: the lower group of HTC will first have a slight deformation, and it will then collapse and maintain a large periodic deformation and finally transition into a periodic small deformation. The upper group will have a slight deformation and maintain a small periodic deformation at first, after which it will collapse and transition into a large periodic deformation.
(5) The numerical simulation results show a nearly identical variation law of the deformation morphology and the curve trend compared with DOFS monitoring. The broken rules obtained using both research methods confirm the feasibility of DOFS monitoring. Moreover, by making the conversion using the geometric similarity constant, the collapse distances of both groups of conglomerates obtained by both methods are shown to be nearly the same, which further verifies the accuracy of the deformation pattern and movement law.

## Figures and Tables

**Table 1:** Research on the deformation of the HTC stratum.

Authors	Main Contribution	Key Research Method
Wang, L. et al. (2009) [22]	Studied the correlation of the ground surface subsidence characteristics and how it induces mining disasters under the HTC	Field monitoring with surface rock movement observation network
Ren, W. et al. (2010) [23]	Analyzed the characteristics of the deformation and failure of the ground surface under the thick overlying terrane	Physical modeling experiment with close-range photogrammetry measurement
Ma, L. et al. (2012) [24]	Analyzed the characteristics of the collapse of HTC, as well as the influence of collapses on the distribution of stress	Numerical simulation
Jiang, F. et al. (2014) [25]	Proposed a prevention and control method for rock bursts in extra-thick coal under the control of HTC and thrust faults	Field monitoring with micro-seismic monitoring
Li, B. et al. (2014) [26]	Demonstrated that the large area of suspended roof subsidence of the HTC will increase the accumulation of static load in the coal seam	Physical modeling experiment with total station displacement monitoring
Chai, J. et al. (2016) [27]	Designed a displacement measurement device to monitor the internal deformation of HTC and proved its feasibility	Physical modeling experiment with internal displacement monitoring
Chai, J. et al. (2018) [28]	Analyzed the floor pressure and rock stress under HTC occurrence by designing a pressure sensor based on DOFS (distributed optical fiber sensor)	Physical modeling experiment with floor pressure sensors based on DOFS system
Xu, C. et al. (2019) [29]	Researched the scale, stress, and energy characteristics of rock bursts under a thick stratum by analyzing the failure process	Numerical simulation and field monitoring with micro-seismic monitoring
Chai, J. et al. (2020) [30]	Investigated the movement of HTC and ground surface subsidence characteristics by considering it as a key stratum	Physical modeling experiment with DOFS strain monitoring and numerical simulation
Liu, X. et al. (2021) [31]	Designed three-zone presplitting blasting technology to study the stability of surrounding rock mass mining under the HTC	Field monitoring with hydraulic support monitoring

**Table 2:** Engineering geology conditions of the overburden stratum that contains HTC rock.

Lithology	Actual Thickness (m)	Modeling Thickness (cm)	Accumulated Modeling Thickness (cm)
Clay	15.0	3.8	201.8
Limestone	5.0	1.3	198.0
Sandstone	65.0	16.3	196.8
Fine sandstone	85.0	21.3	180.5
Conglomerate	250.0	62.5	159.3
Shattered rock	1.0	0.3	96.8
Conglomerate	160.0	40.0	96.5
Mudstone	50.0	12.5	56.5
Fine sandstone	40.0	10.0	44.0
Siltstone	70.0	17.5	34.0
Fine sandstone	25.0	6.3	16.5
Mudstone	25.0	6.3	10.3
Coal	25.0	6.0	6.0
Sandstone	8.0	10.0	20.0
Mudstone	72.0	10.0	10.0

**Table 3:** Mechanical properties of the overburden strata applied to numerical modeling.

Lithology	Actual Thickness (m)	Compressive Strength (MPa)	Tensile Strength (MPa)	Elastic Modulus (×10^3^ MPa)	Density (t/m^3^)
Clay	15.0	15.0	1.5	5.0	1.6
Limestone	5.0	15.0	1.5	5.0	1.6
Sandstone	65.0	35.0	5.5	32.0	1.8
Fine sandstone	85.0	30.0	4.0	28.0	1.6
Conglomerate	250.0	75.0	5.5	32.0	1.7
Shattered rock	1.0	40.0	4.0	28.0	1.7
Conglomerate	160.0	75.0	5.5	32.0	1.8
Mudstone	50.0	50.0	1.2	5.0	1.9
Fine sandstone	40.0	40.0	9.0	35.0	1.6
Siltstone	70.0	45.0	4.0	28.0	1.7
Fine sandstone	25.0	40.0	9.0	31.0	1.6
Mudstone	25.0	50.0	1.2	5.0	1.9
Coal	15.0	16.0	0.6	3.5	0.9
Sandstone	8.0	65.0	5.5	32.0	1.8
Mudstone	72.0	50.0	1.2	5.0	1.9

## Data Availability

The data that support the findings of this study are available from the corresponding author upon reasonable request.
